# Experimental Study on Dispersion Effects of F (1,1) Wave Mode on Thin Waveguide When Embedded with Fluid

**DOI:** 10.3390/s21020322

**Published:** 2021-01-06

**Authors:** Nishanth Raja, Krishnan Balasubramaniam

**Affiliations:** Centre for Non-Destructive Evaluation, Department of Mechanical Engineering, Indian Institute of Technology Madras, Chennai 600 036, India; balas@iitm.ac.in

**Keywords:** ultrasonics, guided wave, waveguide sensors, level sensing, FEM

## Abstract

This paper reports the simultaneous generation of multiple fundamental ultrasonic guided wave modes L(0,1), T(0,1), and F(1,1) on a thin wire-like waveguide (SS-308L) and its interactions with liquid loading in different attenuation dispersion regimes. An application towards liquid level measurements using these dispersion effects was also demonstrated. The finite element method (FEM) was used to understand the mode behavior and their dispersion effects at different operating frequencies and subsequently validated with experiments. In addition, the ideal configuration for the simultaneous generation of at least two modes (L(0,1), T(0,1), or F(1,1)) is reported. These modes were transmitted/received simultaneously on the waveguide by an ultrasonic shear wave transducer aligned at 0°/45°/90° to the waveguide axis. Level measurement experiments were performed in deionized water and the flexural mode F(1,1) was observed to have distinct dispersion effects at various frequency ranges (i.e., >250 kHz, >500 kHz, and >1000 kHz). The shift in time of flight (TOF) and the central frequency of F(1,1) was continuously measured/monitored and their attenuation dispersion effects were correlated to the liquid level measurements at these three operating regimes. The behavior of ultrasonic guided wave mode F(1,1) when embedded with fluid at three distinct frequency ranges (i.e., >250 kHz, >500 kHz, and >1000 kHz) were studied and the use of low frequency Regime-I (250 kHz) for high range of liquid level measurements and the Regime-II (500 kHz) for low range of liquid level measurements using the F(1,1) mode with high sensitivity is reported.

## 1. Introduction

Waveguide based sensors have been used for wide range of measuring applications such as temperature, rheology, fluid level, etc., of the surrounding fluid [1,2,3,4,5,6,7,8,9,10]. Ultrasonic guided waves have been a relevant tool for non-destructive testing (NDT) and structural health monitoring (SHM) technologies due to their wide screening of the acoustic field and their ability to propagate long distances in large structures [9,10,11,12,13]. Ultrasonic guided wave-based waveguide sensors have the potential to resolve some of the major drawbacks of standard temperature (e.g., sensor drift, junction failure, etc.) and level sensors (e.g., splashing, temperature effects, etc.) [1,2,3]. Ultrasonic guided wave-based waveguide sensors have also been reported to measure the physical properties of fluid media (such as mold slags, molten glass, viscous fluids, level, etc.) [2,4,5,6,7,8,9,10] and temperature-dependent elastic modulus [14] extensively. Later, different waveguide configurations such as helical, spiral, and bend waveguides [15,16,17,18] were developed for the distributed temperature measurement [18,19,20] of the surrounding medium. The majority of cylindrical waveguide sensors were designed for either longitudinal L(0,1) [19,20,21], torsional T(0,1) [22,23,24], or flexural wave modes F(1,1) [25,26,27,28].

**Figure 1:**
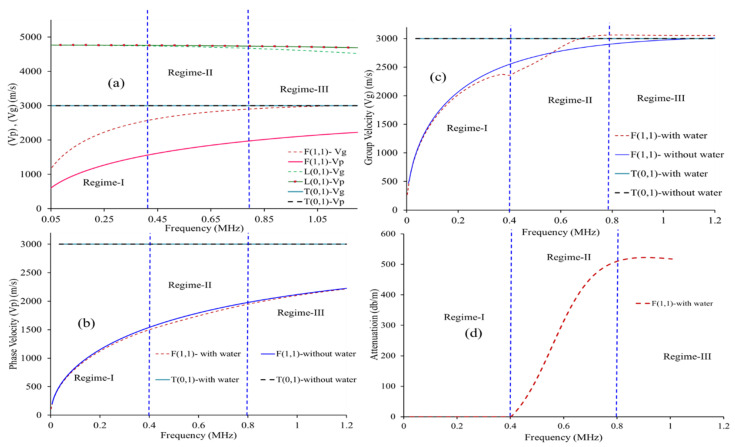
(**a**) Dispersion curves of 1.00 mm dia SS-308L waveguide in free boundary conditions (phase velocity (Vp) and group velocity (Vg)), [(**b**,**c**)] Vp and Vg at fluid (water) loaded, (**d**) Attenuation plot in water loaded condition.

Suresh et al. [14,20] reported three configurations (0°, 45°, and 90°) between the cylindrical waveguide (circular cross-section) and conventional shear transducer for generating different wave modes and concluded that 45° is ideal for the simultaneous generation of two modes (L(0,1) and T(0,1)). He further explored this technique for simultaneous elastic moduli (E&G) measurement at elevated temperatures. The 45° orientation was further explored and the possibility of transmitting/receiving the fundamental L(0,1), T(0,1), and F(1,1) modes simultaneously in pulse-echo mode [28]. This paper analyzes the dispersion behavior of F(1,1) at three attenuation dispersion regimes (refer to Figure 1) in detail using finite element models and experiments. Furthermore, this effect has also been shown to be applied to waveguide-based fluid level measurements.

Finite element method (FEM) studies used ABAQUS 6.12 [29] to study the generation/reception of at least two modes (L(0,1), T(0,1), or F(1,1)) simultaneously using 0°/45°/90° orientation of the shear vibration with respect to the transducer. The optimum angle for obtaining the maximum displacement of the F(1,1) mode and its dispersion effect on a straight waveguide in air media and its behavior at varying central frequencies (250 kHz, 500 kHz, and 1000 kHz) have been studied. Experiments in liquid level measurement were also performed from 0 to 100 mm using deionized water (inviscid fluid) to understand the nature of F(1,1) under fluid loading conditions. The detailed FEM observations as well as experimental results are reported in the Secs III and IV.

A trade-off between the required range of level measurement and the resolution of the level sensing in process industries may be controlled by selecting the appropriate operating regime based on the specific application. Additionally, it is also possible (although not in the scope of this manuscript) by simultaneously generating other wave modes viz. either L(0,1) or T(0,1), additional physical properties such as temperature and/or rheology of the surrounding fluid can be measured.

## 2. Background

### 2.1. Guided Wave in Cylindrical Waveguides

In cylindrical waveguides, three-mode families, longitudinal (L), flexural (F), torsional (T), exist, and the propagation of cylindrical guided waves in a waveguide is characterized through their material properties, length, frequency, and phase (Vp)/group velocity (Vg). In this study, a stainless-steel wire of 1.00 mm thick was selected as the waveguide on the basis of its non-corrosive nature and its material properties are shown in Table 1. The phase and group velocity dispersion curve of 1.00 mm SS-308L waveguide and their corresponding attenuation plots were obtained using Disperse [30] and shown in Figure 1. It is worth noting that the dispersion effects are observed primarily for the F(1,1) mode in the frequency range. From Figure 1d, the attenuation behavior of F(1,1) can be categorized into three regimes:

Regime-I: 0–400 kHz where the attenuation is very small due to the absence of leakage into the fluid. In this regime, the phase velocity of the guided wave is below the fluid velocity.

Regime-II: 400–800 kHz where the attenuation is rapidly increasing (i.e., until 500 dB/m).

Regime-III: above 800 kHz where the attenuation if very high (i.e., above 500 dB/m). In this region, the phase velocity of the F(1,1) matches the shear velocity of waveguide material.

### 2.2. FEM Simulation Studies

**Figure 2:**
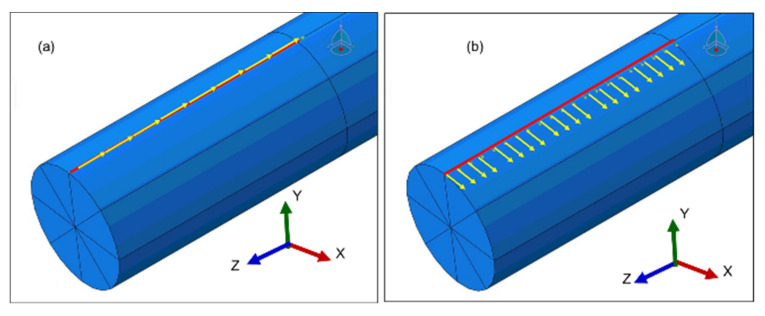
Snapshot of FEM of the cylindrical waveguide and the input loading. (**a**) Parallel and (**b**) perpendicular to the waveguide axis.

The FEM analysis was carried out to find an optimal angle of excitation for simultaneously transmitting and receiving at least two fundamental guided wave (GW) modes (L(0,1) and/or T(0,1) and/or F(1,1)) and to study their mode behavior in straight waveguide configuration when embedded in air and inviscid fluid (de-ionized water) at different immersion rates. The FEM and waveguide parameters are shown in Table 1. Figure 2a shows the snapshot of the FEM model with a cylindrical waveguide with an input signal displacement at the surface of the waveguide oriented along the waveguide axis and Figure 2b shows the input loading perpendicular to the waveguide axis.

**Figure 3:**
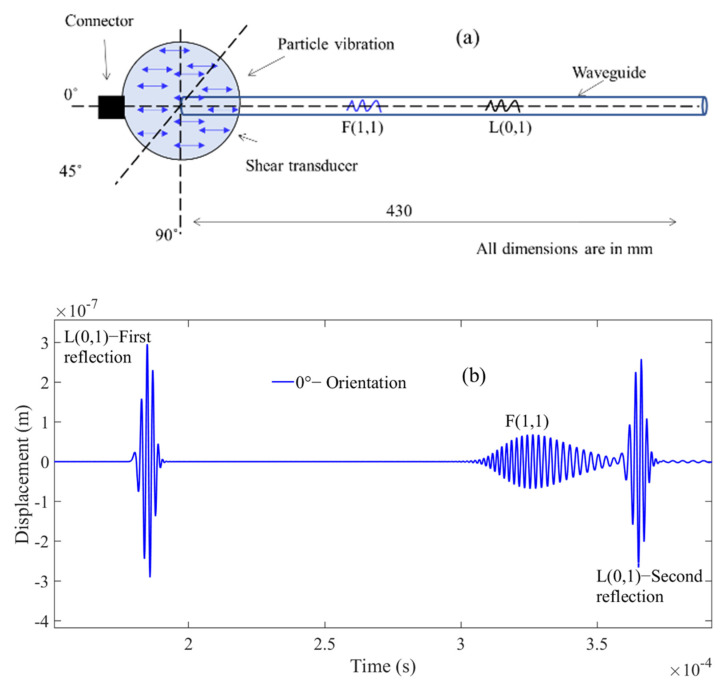
(**a**) Shows the shear transducer orientation (0°) to the waveguide axis (**b**) and the reflected A-Scan signals from FEM simulations with L(0,1) and F(1,1).

**Figure 4:**
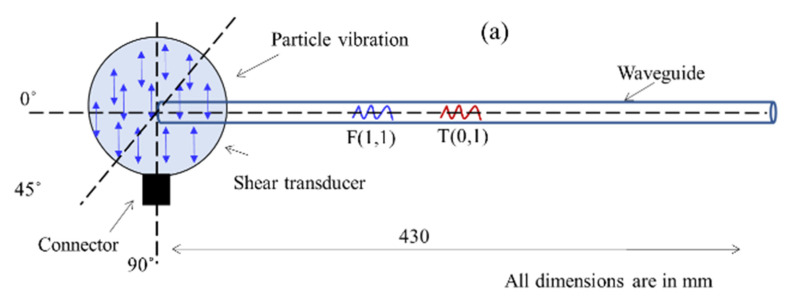

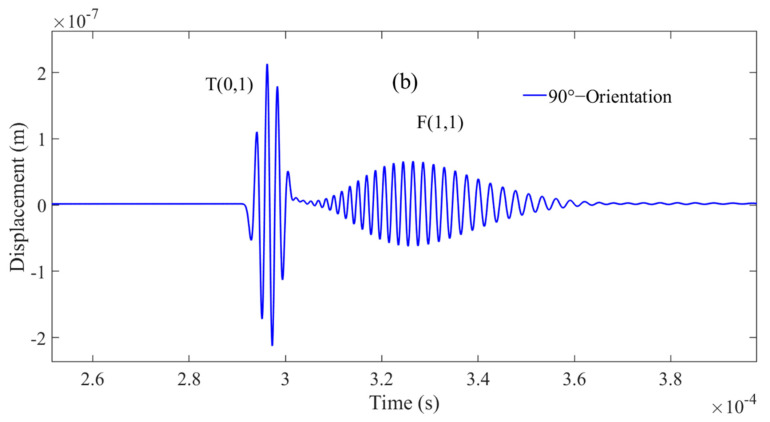
(**a**) Shows the shear transducer orientation (90°) to the waveguide axis (**b**) and the reflected A-Scan signals with T(0,1), and F(1,1).

This configuration is schematically shown in Figure 3a which shows the shear transducer with three possible surface traction configurations (0°, 45°, and 90° w.r.t axis of the cylindrical waveguide). Figure 3a shows the A-Scan signals of a straight waveguide with an input signal displacement at the surface of the waveguide oriented along the waveguide axis (0°). Figure 4a presents the angle of excitation perpendicular to the axis of the waveguide (90°).

**Figure 5:**
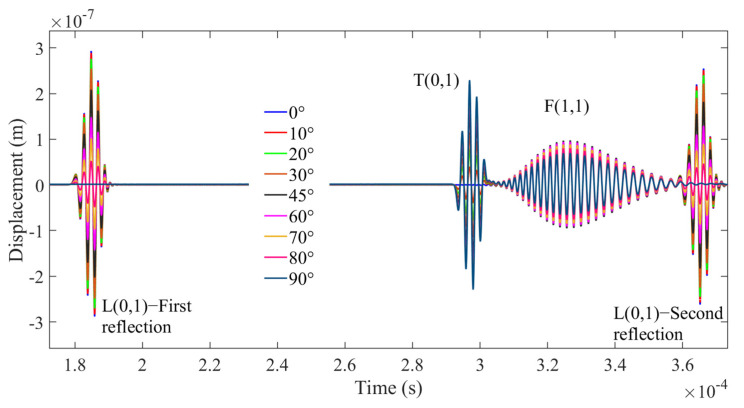
Signals acquired at different angles of excitation (0°–90°).

Figure 3b and Figure 4b show that at least two wave modes are simultaneously excited/received L(0,1) and F(1,1) at 0° configuration and T(0,1) and F(1,1) at 90°. In addition, FEM studies have also been conducted at a different angle of excitation from 0° to 90° to find the optimum angle to achieve the maximum displacement of the F(1,1) mode. The obtained A-Scan signals are shown in Figure 5, it was observed that the 45° excitation provides the maximum displacement of the F(1,1) mode and 0° configuration for L(0,1) and 90° for T(0,1), respectively.

**Figure 6:**
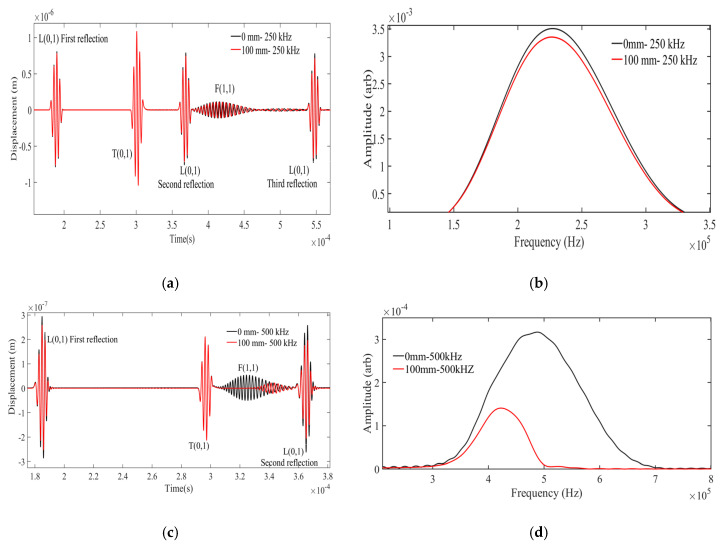

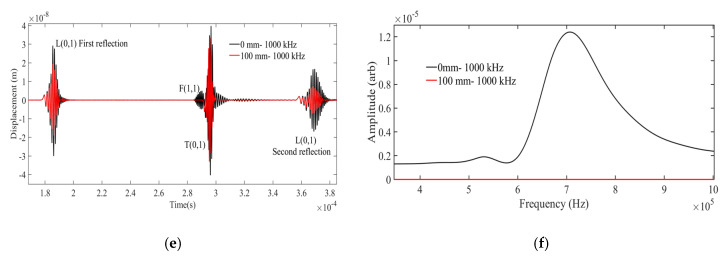
A-Scan signals at the frequencies of (**a**) 250 kHz, (**c**) 500 kHz, and (**e**) 1000 kHz at a 45° angle of excitation and their corresponding FFT plots of F(1,1) at (**b**) 250 kHz, (**d**) 500 kHz, and (**f**) 1000 kHz.

The FEM simulations were further continued with the 45° excitation and the L(0,1), T(0,1), and F(1,1) modes were studied. Here, the wire-like waveguide was loaded with water (e.g., 0 mm, i.e., air medium and 100 mm, i.e., water) at different central frequencies (Regime-I (250 kHz), Regime-II (500 kHz), Regime-III (1000 kHz)). In order to compare the relative behavior of the three modes in the three regimes using the FEM results, the A-Scan for three different frequencies viz. 250 kHz, 500 kHz, and 1000 kHz for a fluid loading of 100 mm was analyzed and summarized in Table 2. The obtained A-Scan signals are illustrated in Figure 6a,c,e and the corresponding time-gated Fast Fourier Transform (FFT) of the F(1,1) mode is shown in Figure 6b,d,f. Based on the dispersion curves provided in Figure 1, the following observations can be inferred from Figure 6.

(a) The F(1,1) mode is relatively more sensitive to the surrounding inviscid fluid media compared to L(0,1) and T(0,1) across the three regimes.
(b) The L(0,1) wave mode remains non-dispersive at 250 kHz and 500 kHz and becomes dispersive when operated at 1000 kHz.
(c) T(0,1) remains non-dispersive across these regimes.
(d) In the low attenuation region (Regime-I), the F(1,1) mode exhibits significant time of flight (TOF) shift (refer to Figure 6a) and negligible changes in signal amplitude and peak frequency (refer to Figure 6b)
(e) In Regime-II, significant amplitude drop and TOF shift was noticed in F(1,1) modes (refer to Figure 6c) and notable peak frequency shift were observed (Figure 6d).
(f) It was noticed in Regime-III that the attenuation (amplitude drop) of the F(1,1) mode is predominant when compared to the TOF and frequency shift (refer to Figure 6c). The velocity of F(1,1) also matches with the T(0,1) velocity; hence, the extraction of FFT of (F1,1) is carried out in the region marked in Figure 6e.
(g) Additionally in the Regime-III, the peak frequency of F(1,1) is around 700 kHz (refer to Figure 6f).
(h) All three wave modes follow the dispersion curve results across these regimes.

The FEM results of the F(1,1) mode are validated using experiments and reported in detail in the next section.

## 3. Experimental Setup and Validation

**Figure 7:**
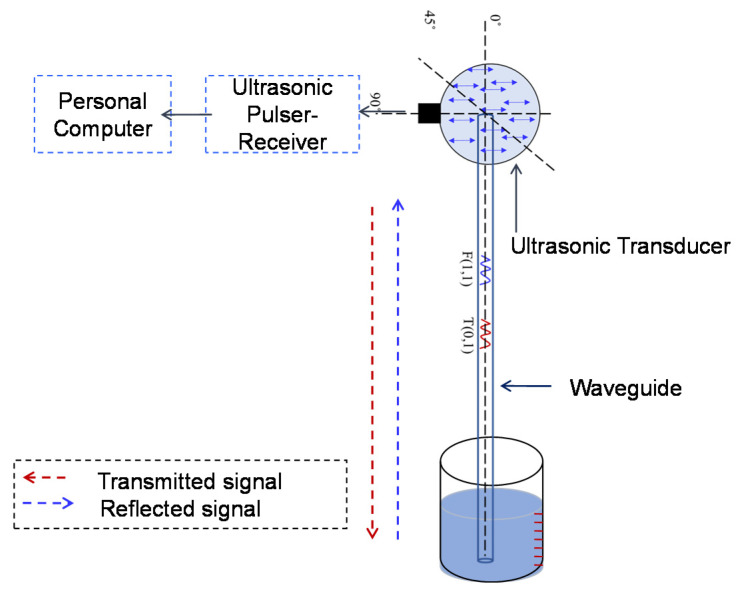
Experimental setup schematic for level measurement experiments.

The previously reported experimental setup [28] was used for level measurement experimentation and the device schematic is shown in Figure 7. During experiments, the 1 mm stainless steel waveguide (SS-308L) was vertically positioned and the shear transducer was attached at one end and the other end was immersed in the liquid container (with 0–100 mm range and 1 mm accuracy) for level measurement trials. The PZT based shear transducer (Panametrics V150—250 kHz/V151–500 kHz/V153–1000 kHz) was attached at an angle (90°) to the surface of the waveguide as shown in Figure 7 to transmit/receive the desired wave modes (T (0,1) and F (1,1)) simultaneously in pulse-echo mode. This 90° arrangement was investigated for fluid level measurement, and their response to various frequency ranges (viz. 250 kHz, 500 kHz, and 1000 kHz) was examined. To transmit and receive the signals an ultrasonic pulser/receiver (Olympus NDT PR 5077 Pulser/Receiver) and a DAQ (Picoscope 5242D) with a sampling rate of 400 MHz was used. A very thin film of an ultrasonic couplant (viscous silicone) was used between the waveguide and the transducer to establish proper contact and avoid the air gap. Using a mechanical fixture, the normal force between the waveguide and the transducer was optimized in order to maximize the amplitude of the F(1,1) mode using trial-and-error. This configuration was used for all experiments reported here.

## 4. Results and Discussion

**Figure 8:**
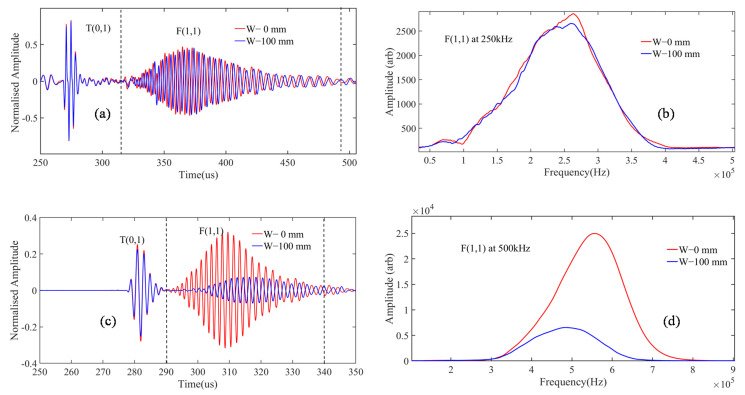

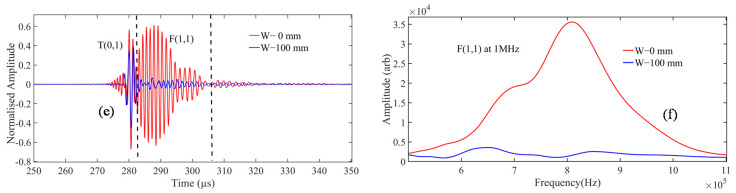
Obtained A-Scan signal at 0 cm and 10 cm fluid loading at the frequencies of (**a**) 250 kHz, (**c**) 500 kHz, and (**e**) 1000 kHz and their corresponding FFT plots at (**b**) 250 kHz, (**d**) 500 kHz, and (**f**) 1000 kHz.

The initial A-Scan signal was acquired with the waveguide in air medium (0 mm), and in an inviscid fluid medium (water, 100 mm) and their corresponding A-Scan signals at different frequencies regimes are shown in Figure 8a,c,e. The F(1,1) wave mode shows sensitivity to change in inviscid fluid level in both TOF and amplitude, while the T(0,1) mode shows only change in amplitude. The F(1,1) mode is preferred over the T(0,1) mode because the TOF measurements are more reliable compared to the amplitude-based measurements. In addition, the relative change in amplitude for the F(1,1) mode is significantly more when compared to the T(0,1) mode in Regime-II (500 kHz) and III (1000 kHz).

Subsequently, using time gates, the F(1,1) modes were evaluated in the time domain and their frequency spectrums were plotted and shown in Figure 8b,d,f. From the experimental results, the following observations were made of the F(1,1) mode:

- At 250 kHz (Regime-I), negligible change in peak frequency and signal leakage to the surrounding medium is minimal, but significant change in TOF was noted.
- Whereas in 500 kHz (Regime-II), significant change in TOF and peak frequency shift and signal leakage to the surrounding medium was noted. This is mainly due to the attenuation dispersion effects of F(1,1) at this operating frequency regime.
- Finally, at Regime-III (1000 kHz), the attenuation and the wave leakage of F(1,1) to the fluid medium is higher when related to Regime-I and -II and merges with the T(0,1), since both the F(1,1) and T(0,1) velocity matches at this operating frequency.
- It was also noticed at Regime-III (1000 kHz) that the frequency of the F(1,1) mode was around 800 kHz.

**Figure 9:**
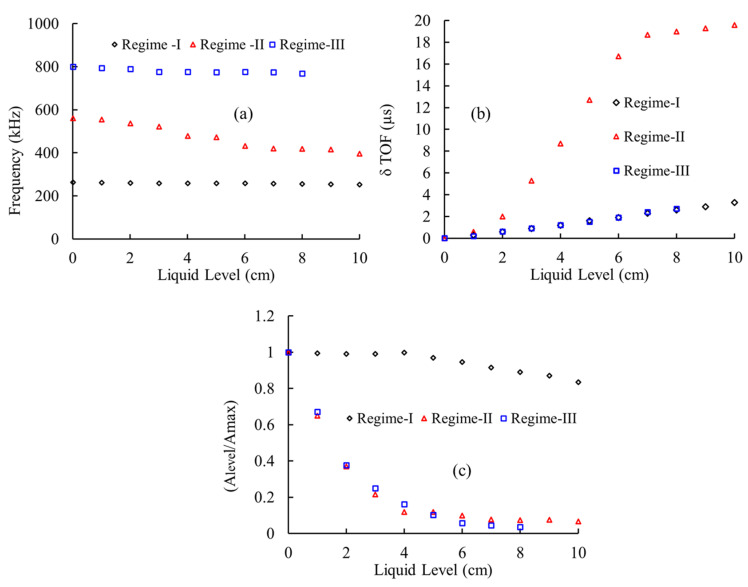
Illustrates the (**a**) shift in peak frequency, (**b**) change in time of flight (δTOF), and (**c**) drop in amplitude of F(1,1) wave modes at different fluid levels in Regime-I (250 kHz), Regime-II (500 kHz), and Regime-III (1000 kHz).

The response of F(1,1) at each level of immersion are shown in Figure 9. It was observed that at 250 kHz (Regime-I), the TOF shift was found to be optimum for level sensing, whereas in 500 kHz (Regime-II), both the change in TOF and peak frequency shift were found to be higher. Finally, at Regime-III (1000 kHz), the drop in amplitude and the change in TOF were found to be optimal for level measurement.

The experimental results match with the FEM observations. However, owing to its better sensitivity/dispersive nature, the F(1,1) mode is appropriate for accurate level sensing and effective for a wide-range of level measurement, i.e., to measure the level with high accuracy and sensitivity. Regime-II is preferred (TOF shift—(0.196 µs/mm), frequency shift—(1.6 kHz/mm), and amplitude drop—(0.067/mm)); however, the range of measurement is limited to only to 100mm. Nevertheless, if the range of measurement is higher than 100mm, then Regime-I is preferred (TOF shift—(0.029 µs/mm), frequency shift—(0.1 kHz/mm) and amplitude drop—(0.083/mm)).

In contrast, the L(0,1) and T(0,1) modes exhibit limited sensitivity/dispersive natures and could be employed for very long-range level sensing trials. However, the choice of the wave mode and its optimum operating frequency would rely on the application.

## 5. Repeatability Experiments

**Figure 10:**
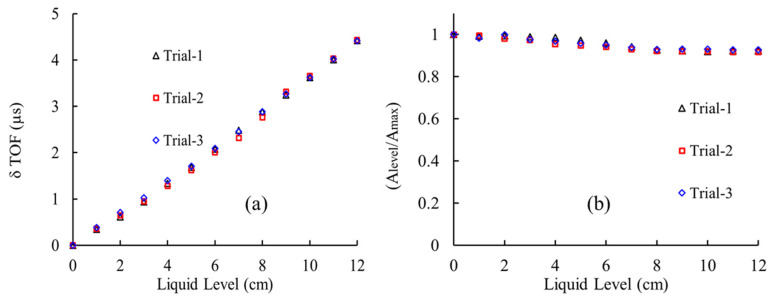
(**a**) Change in time of flight (δTOF) and (**b**) drop in amplitude of F(1,1) wave modes at different fluid levels in Regime-I (250 kHz) for different trials.

The level measurement experiments were repeated using a 1mm waveguide (SS-308L) with an operating frequency of 250 kHz (Regime-I) for multiple trials to validate the repeatability of this technique. The range of measurement was fixed at 12 cm and the results were obtained at each level of immersion. From the earlier results, it was observed that the change in time of flight (δTOF) and drop in amplitude was found to be dominant in Regime-I; hence, the response of F(1,1) at each level of immersion are monitored and shown in Figure 10. From Figure 10, it is evident that the data was found to be consistent and confirmed to be repeatable with an error percentage less than 2.5%. The obtained results follow the same pattern of the previous results reported in Figure 9. Combined with a calibrated curve of the δTOF against the liquid level, it will be possible for monitoring the rapid changes in fluid level with more precision and accuracy.

## 6. Conclusions and Future Work

This paper reported the optimum configuration for simultaneous generation/reception of at least two fundamental guided wave mode (L(0,1), T(0,1), or F(1,1)) using 0°–90° orientation to the axis of the waveguide. Subsequently, the dispersive effects of L(0,1), T(0,1), and F(1,1) were studied at multiple dispersion attenuation regimes (Regime-I (250 kHz), Regime-II (500 kHz), and Regime-III (1000 kHz)). The results obtained from FEM and the experimental results confirm the possibility of using a waveguide sensor based on the F(1,1) mode for applications such as liquid level measurement. The key contribution of this paper are:

- The use of highly sensitive F(1,1) mode-based level sensing approach that has not been reported elsewhere.
- The exploitation of F(1,1) at three distinct frequency ranges (i.e., >250 kHz, >500 kHz and >1000 kHz) was studied and validated by FEM and experimental results.
- The use of Regime-I for higher range of measurements and Regime-II for lower range of measurements with high sensitivity is discussed.

The liquid level can be estimated by tracking the major variations in TOF (Regime-I (0.032 us/mm), Regime-II(0.196 us/mm), and at Regime-III (0.033 us/mm)) and frequency shift (Regime-I (0.1 kHz/mm), Regime-II (1.6 kHz/mm), and at Regime-III (0.4 kHz/mm)). Taking advantage of the F(1,1) mode’s three regime behavior, our recommendation is as follows,

- For measurement of level with high sensitivity but low range, the Regime-II is preferred. For example in the case demonstrated an excellent sensitivity in TOF shift—(0.196 µs/mm) and Frequency shift—(1.6 kHz/mm), can be achieved, however the range of measurement is limited only to 100mm approximately.
- For measurement of level with lower sensitivity but higher range, the Regime-I is preferred. For example in the case demonstrated an acceptable sensitivity in TOF shift—(0.029 µs/mm) and frequency shift—(0.1 kHz/mm). However the range of measurement can be extended to more than 1000 mm.

Our results demonstrate that TOF shift is the best indicator for F(1,1) based level sensing and this is a versatile technique for accurate level measurement in various complex industrial environments and applications. During industrial integration, more care should be taken to build a fixture that is more robust in order to rigidly connect the waveguide to the transducer and to preserve the alignment between the transducer and the waveguide axis constantly. This work can be further extended to simultaneously measure the fluid temperature and level, where the fluid temperature can be calculated using one wave mode while the level can be estimated using the other wave mode.

## Figures and Tables

**Table 1:** Finite element method (FEM) and waveguide parameters.

Material	Stainless Steel
Grade	308 L
Waveguide Diameter (D)	1.00 mm
Mass density (ρ)	7932.00 kg/m^3^
Young’s modulus (E)	183.00 GPA
Poisson’s ratio (µ)	0.30
Number of cycles	5
Central frequencies (kHz)	250, 500, 1000

**Table 2:** Response of three-wave mode to inviscid fluid loading at different operating frequency regimes.

Operating Frequency	Frequency Shift	TOF Shift	Amplitude Drop
Regime-I	F(1,1)	F(1,1)	L(0,1), F(1,1)
Regime-II	F(1,1)	F(1,1)	L(0,1), F(1,1)
Regime-III		F(1,1)	L(0,1), F(1,1)

## Data Availability

The data presented in this study are available on request from the corresponding author.
